# Bilateral Paramedian Thalamic Infarction Initially Presenting as a Convulsive Seizure

**DOI:** 10.1155/2013/704952

**Published:** 2013-05-23

**Authors:** Jianping Wang, Xiaojie Fu, Chao Jiang, Hengfang Liu, Yuanzheng Zhao, Wei Han

**Affiliations:** Department of Neurology, The Fifth Affiliated Hospital of Zhengzhou University, Henan, Zhengzhou 450052, China

## Abstract

Bithalamic infarctions initially presenting as a convulsive seizure are rarely reported and, to our best knowledge, have never been reported in China. Here, we present a patient with convulsive seizure at the onset of bilateral paramedian thalamic infarction. The diffusion-weighted imaging revealed that the infarct area is supplied by Percheron artery. Associated with the relationship between seizure and centrencephalic system and reticular formation as previously reported, we suggest that seizure could be the onset symptom of paramedian thalamic infarction. Physicians should recognize this condition, because both seizure control and early ischemic stroke management are required.

## 1. Introduction

Bithalamic infarctions represent 0.6% of ischemic stroke [1]. The anatomic etiology is presumed to be the occlusion of Percheron artery, an uncommon vascular variation, in which a single common trunk from one of the P1 segments of the posterior cerebral artery provides bilateral irrigation to the paramedian thalami [2]. Bilateral paramedian thalamic infarctions initially presenting as a convulsive seizure are rarely reported. The mechanism of onset seizure is not clear but may be related to the lesions of centrencephalic system as well as the reticular formation. We are reporting, to our best knowledge, the first bilateral paramedian thalamic infarct case initially presenting as seizure in China.

## 2. Case Report

**Figure 1:**
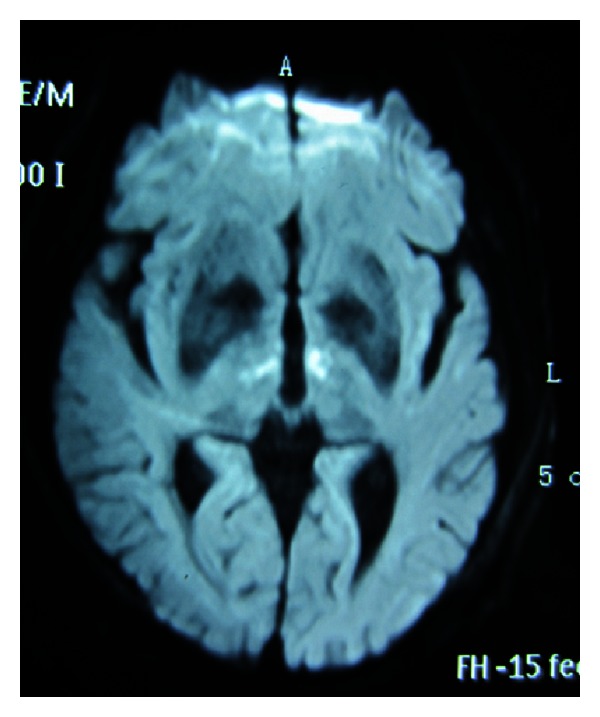
Diffusion-weighted imaging obtained until 48 hours after the onset of symptoms showed high signal intensity bilaterally in paramedian thalami.

**Figure 2:**
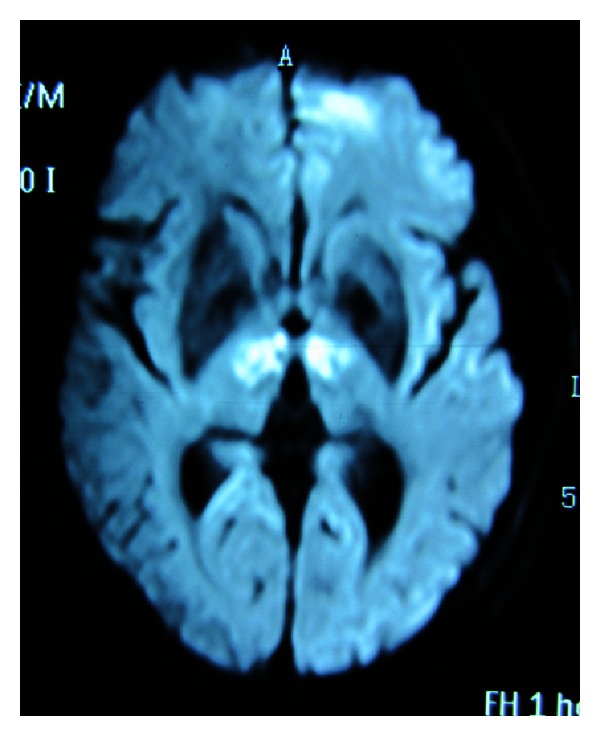
Diffusion-weighted imaging performed six days after the admission showed a larger area of high signal intensity bilaterally in paramedian thalami than before.

A 66-year-old man, with two-year atrial fibrillation, ischemic stroke, and thirty-year smoking history, was admitted to the emergency department of our hospital. He was found to be unconscious and had bruised tongue and urinary incontinence after about 10 s clonic movements of all four limbs beside his bed in the morning. On admission, his blood pressure was 144/110 mmHg and irregular heart rate was 84 beats/min; his pulse was 78 beats/min. The neurological examination revealed that the patient was comatose and his bilateral Babinski signs were positive. Urgent computed tomography (CT) performed two hours after the onset of symptoms showed lacunar infarction in bilateral basal ganglia. Glucose levels were normal. After CT scan, aspirin (100 mg/d) and atorvastatin (20 mg/d) were used for his treatment. Then he was admitted to the neurological ward. Owing to the patient's lack of cooperation, an MR image could not be obtained until 48 hours after the onset of symptoms. Trace diffusion-weighted imaging (DWI) showed bilaterally high signal intensity in paramedian thalami (Figure 1), and the restriction of water diffusion was confirmed on the apparent diffusion coefficient (ADC) maps. Magnetic resonance venography (MRV) results were normal. Electrocardiography (ECG) indicated atrial fibrillation; BNP was 303 pg/mL and TSH was 0.338 uIU/L. The results of the following tests were normal: blood cell count, arterial blood gas, electrolyte level, and cerebrospinal fluid. Echocardiogram and electroencephalogram (EEG) results were also normal. Five days after admission, he became conscious but sleepy throughout the day. He did not take the initiative to speak but could answer simple questions in a whisper with a few incorrect words. The vertical gaze paresis was observed in this patient. Follow-up DWI performed six days after the admission showed a larger area of high signal intensity bilaterally in paramedian thalami than before (Figure 2). During the following week the patient experienced drowsiness accompanied with restlessness and he showed childish attitude and aggressiveness. He could not recognize his wife when he was transferred to the rehabilitation ward twenty days after his admission.

## 3. Discussion

Onset seizure is rarely observed in bilateral paramedian thalamic infarction [3] and, to our best knowledge, has never been reported in China.

Bithalamic infarctions are infrequently reported and represent 0.6% of ischemic stroke [1]. The anatomic etiology is presumed to be the occlusion of the artery of Percheron, an uncommon vascular variation, in which a single common trunk from one of the P1 segments of the posterior cerebral artery provides bilateral irrigation to the paramedian thalami [2]. Bithalamic infarctions can cause the bilateral ventromedial thalamic syndrome (BVTS). The BVTS is characterized by the following elements: decreased arousal, mood changes, vertical gaze paresis, and memory difficulties [4], which are in accordance with our case. 

Early-onset seizure is thought to be caused by ischemic or hemorrhagic lesions in the cerebral cortex [5]. The mechanism is unknown but may be related to the acute focal metabolic derangement including local acidosis, brain edema, and altered electrolyte balance as well as neurotransmitter activity [6]. In general, early-onset seizure caused by cerebral infarcts is relatively more common when the anterior circulation, rather than the posterior circulation, is affected [7]. The mechanism of onset-seizure caused by the infarction in the posterior circulation is not clear but maybe related to the lesions of “centrencephalic system,” which are the neurons in the central core of brainstem from the thalamus to the medulla oblongata, connecting the cerebral hemispheres. Penfield suggested this system functioned as a causative center of seizures [8]. In addition, animal experimental studies have suggested that electrolytic lessoning of reticular formation (RF) also can cause seizure [9]. So the infarct lesions of Bithalamic, which are part of the centrencephalic system and the reticular formation, can induce seizure as well as unconsciousness.

Giroud reported that 16.6% of stroke cases caused by cardiogenic embolus would have seizure within 15 days [10]. In this case, the patient has no other risk factors except two-year history of atrial fibrillation without taking any specific medicines and thirty-year smoking history. We conclude that cardiogenic embolus is responsible for the occlusion of Percheron artery and the bilateral paramedian thalamic infarction. In addition, the damage of the centrencephalic system and the reticular formation may be related to the syndrome of seizure and unconscious.

In summary, seizure could be the initial symptom of bilateral paramedian thalamic infarction. Physicians should recognize this condition, because not only seizure control but also early ischemic stroke management is required.
